# Effects of Traditional and Technology-Based Exercise Interventions on Cognitive Function in Older Adults: A Systematic Review and Meta-Analysis

**DOI:** 10.3390/jintelligence14080177

**Published:** 2026-08-01

**Authors:** Jingzhan Ren, Xiaotong Du, Wei Wang, Sijing Fan, Han Wang, Gongxing Fang, Yuan Li, Ruilong Wang, Hegui Bao, Chenyu Zhang, Ruohan Zhu, Xinming Ye, Wen Fang

**Affiliations:** 1 School of Business, East China University of Science and Technology, Shanghai 200237, China; 2 School of Sports Science and Engineering, East China University of Science and Technology, Shanghai 200237, China; 3 Faculty of Digital Creativity, Anhui Sanlian University, Hefei 230601, China; 4 School of Law, Zhejiang University of Finance and Economics, Hangzhou 310018, China

**Keywords:** older adults, cognitive function, exercise interventions, technology-assisted exercise, network meta-analysis

## Abstract

Background: As population aging accelerates, pharmacological treatments provide limited benefits for cognitive decline. Exercise and technology-assisted exercise have thus emerged as important non-pharmacological approaches for supporting cognitive health in older adults. However, comparative evidence on the relative effectiveness of different intervention modalities across cognitive outcomes remains limited. Objective: This study used a network meta-analysis to systematically compare and rank the effects of traditional and non-traditional exercise interventions on cognitive function in older adults. Methods: This systematic review and network meta-analysis was registered on PROSPERO (CRD420261278685). PubMed, Embase, the Cochrane Library, Web of Science, and Scopus were searched from inception to May 2026. Randomized controlled trials enrolling participants aged 60 years or older were included. Interventions comprised aerobic, resistance, mind–body, finger, and multicomponent exercise, as well as virtual reality-based interventions, artificial intelligence-assisted exercise, and wearable exoskeleton training. Primary outcomes included global cognition, executive function, memory, attention, and activities of daily living. A random effects network meta-analysis was applied to estimate standardized mean differences with 95 percent confidence intervals, and intervention rankings were derived using SUCRA values. Results: A total of 70 randomized controlled trials involving 5573 older adults were included. All active interventions demonstrated overall cognitive benefits compared with usual care or non-active control conditions. Mind–body exercise showed the highest probability of improving global cognitive performance. Virtual reality-based interventions were particularly effective for executive function, memory, and activities of daily living, while artificial intelligence-assisted exercise showed favorable rankings for attention outcomes. Mind–body exercise showed favorable effects on global cognition, whereas several technology-assisted interventions ranked highly for selected cognitive and functional outcomes. Conclusions: Both traditional and non non-traditional exercise interventions improve cognitive function in older adults, although their effects differ across cognitive domains. Technology-assisted approaches appear more effective for enhancing specific cognitive functions, while traditional interventions such as mind–body exercise are more advantageous for preserving overall cognitive performance. These findings highlight the potential value of stage specific and combined intervention strategies for promoting cognitive health in aging populations.

## 1. Introduction

Population aging is accelerating worldwide and has emerged as one of the most profound social transformations of the twenty first century. Global population aging is projected to accelerate rapidly, with the population aged 65 years and older expected to more than double over the coming decades and reach over 1.5 billion by 2050 (Navaneetham & Arunachalam, 2025). As cognitive abilities gradually deteriorate with age, older individuals face considerable health risks, and society is confronted with an increasingly important public health concern (Sun & Li, 2023). Cognitive impairment directly compromises information processing, decision-making, and adaptive capacity (Alfeo et al., 2024), thereby undermining independent living and increasing reliance on family caregivers and social support systems (Reynolds et al., 2022). The global burden of dementia is projected to increase markedly over the coming decades. An estimated 55.2 million people were affected in 2019, and this population may grow to approximately 78 million by 2030 and more than 150 million by 2050 (Qassem et al., 2023). Dementia also places a heavy financial burden on society. Its global cost was about US$1.3 trillion in 2019. It continues to increase pressure on health and social care systems worldwide (Pedroza et al., 2022).

Cognitive decline is a key precursor and driver of functional impairment in older adults. Problems with memory, attention, and executive function can make daily activities more difficult (Marshall et al., 2011). This may increase the risk of falls, medication mistakes, and poor nutrition (Kupisz-Urbanska & Marcinowska-Suchowierska, 2022; Portlock et al., 2023), and ultimately undermining quality of life (Mitchell et al., 2010). Currently, no approved pharmacological therapy can effectively reverse or halt neurodegeneration-related cognitive decline (Colizzi et al., 2022). These drugs may relieve symptoms, but their effects are usually modest and short-lived (Glynn-Servedio & Ranola, 2017; Ruangritchankul et al., 2021). Importantly, no disease-specific treatment has been established for mild cognitive impairment, a stage that may still allow cognitive remission (Yu et al., 2025). Once dementia becomes clinically manifest, extensive neuronal loss has already occurred, substantially limiting the effectiveness of intervention and narrowing the window for disease modification (van der Flier et al., 2023).

Current drug treatments have limited effects on cognitive function in older adults. Therefore, researchers have paid more attention to non-drug approaches, such as cognitive training and physical exercise. These methods are generally safe, accessible, and may help protect cognitive function (Ribarič, 2022; Smart et al., 2017; Yao et al., 2020). Systematic reviews and meta-analyses show that structured lifestyle and behavioral programs can improve cognitive function in older adults. These benefits include overall cognition and specific cognitive abilities. The improvements can occur without drug treatment (Chib et al., 2025). For example, aerobic exercise has been shown to increase cognitive function in aging populations (Song & Doris, 2019). Network meta-analyses also suggest that several types of exercise can benefit cognitive function in older adults. These include resistance training, aerobic exercise, mind–body exercise, and combined exercise programs (H. Han et al., 2025; Wu et al., 2019).

Meanwhile, technology interventions represented by virtual reality, artificial intelligence, and wearable exoskeletons introduce novel forms of stimulation through human–machine interaction, real time feedback, and immersive task engagement, offering innovative avenues for improving cognitive function in aging population. Systematic reviews and meta-analyses suggest that virtual reality can be useful for cognitive training. Its benefits may be especially relevant for people with mild cognitive impairment whom immersive cognitive rehabilitation may help delay the progression of cognitive decline (Ren et al., 2024; Tortora et al., 2024). Artificial intelligence-assisted exercise has shown promising approach in physical rehabilitation, with potential advantages in accessibility and monitoring (Sumner et al., 2023). In addition, mobile health applications, as accessible non-non-pharmacological interventions, show potential for supporting the maintenance or improvement of cognitive function in aging population with mild cognitive impairment (MCI) or dementia (Chan et al., 2025).

Although existing studies have demonstrated the positive effects of both traditional exercise interventions and technology-assisted interventions on cognitive function in older adults, these approaches have not yet been systematically compared within a unified analytical framework. Most of the current literature evaluates the cognitive effects of traditional exercise or technology-assisted interventions such as virtual reality-based exercise independently from their respective perspectives, with limited direct comparative evidence. As a result, it remains unclear whether technology-assisted exercise interventions confer additional cognitive benefits beyond those of conventional exercise, whether different exercise modalities exert differential effects across specific cognitive domains or subpopulations of older adults, or whether potential synergistic effects may exist between different forms of exercise-based interventions.

Following the PRISMA-NMA guidelines, this study reviewed 70 randomized controlled studies. It compared the effects of traditional exercise and technology-assisted exercise on cognitive function in older adults. By integrating direct or indirect evidence, we compared and ranked different types of exercise. The outcomes included global cognition, executive function, memory, attention, and daily living ability. Building upon previous network meta-analyses, the present study expands the evidence base by incorporating a broader range of emerging technology-assisted exercise interventions and evaluating their relative effectiveness across multiple cognitive domains, thereby clarifying the complementary roles of conventional and technology-assisted exercise interventions in preserving cognitive function among older adults. The findings provide evidence-based guidance for optimizing non-non-pharmacological strategies to support cognitive health in aging populations.

## 2. Methods

### 2.1. Study Design

This study followed the PRISMA-NMA guidelines. The review was registered under CRD420261278685 on 13 March 2026. We conducted a systematic review and network meta-analysis to compare traditional and non-traditional exercise interventions. The main outcomes were measures of cognitive function in older adults.

### 2.2. Literature Search Strategy

Five electronic databases were searched: PubMed, Embase, the Cochrane Library, Web of Science, and Scopus. The final search was completed in May 2026. To enhance search specificity, a comprehensive strategy incorporating detailed terms related to both traditional and non-non-traditional exercise interventions, such as aerobic exercise, resistance training, mind–body exercise, and virtual reality-based interventions, was applied. Keywords related to the target population and cognitive outcomes were combined, including “older adults,” “cognition,” “executive function,” and “memory.” To avoid retrieving irrelevant records, nonspecific terms such as “AI” were not used as standalone keywords. Boolean operators (AND and OR) were used to identify all eligible studies. Google Scholar was also searched to identify eligible studies missed by the five databases. After duplicate removal, the remaining records were imported into ASReview to assist title and abstract screening, while final eligibility decisions were made manually by reviewers according to the predefined criteria. Supplementary Material File S1 contains the complete search strategy.

### 2.3. Inclusion and Exclusion Criteria

Eligibility was determined based on the following criteria: (1) participants aged 60 years or older; (2) interventions involving technology-assisted approaches, including artificial intelligence-assisted exercise, wearable exoskeleton-assisted training, and virtual reality-based training, or traditional exercise modalities, including aerobic exercise, resistance training, mind–body exercise, finger exercise, and multimodal exercise (See Supplementary Materials File S1); (3) randomized controlled trial (RCT) design; and (4) reporting at least one relevant physiological or psychological outcome, including global cognition, executive function, memory, attention, or activities of daily living.

We excluded studies that were not randomized controlled trials. Studies without an exercise or artificial intelligence component were also excluded. Other reasons for exclusion were insufficient data, conference abstracts, and duplicate publications. We also excluded general fitness or health promotion programs that lacked clear rehabilitation goals or standardized intervention protocols. No included studies involved general fitness or preventive programs, as all interventions were explicitly rehabilitation oriented. Detailed criteria for intervention classification are provided in the Supplementary Materials File S1.

### 2.4. Data Extraction and Quality Assessment

Data were collected independently by two reviewers using a standardized form. The extracted data included the first author, publication year, sample size, participant characteristics, intervention type, intervention duration, comparator, outcomes, and assessment tools. When necessary, additional details were obtained from Supplementary Materials. For studies with multiple assessment time points, the first post-intervention measurement was selected for the primary analysis. Any discrepancies in data extraction were resolved through discussion. A third reviewer was involved when needed. Supplementary Material File S2 contains the full dataset used for the network meta-analysis.

### 2.5. Statistical Analysis

Stata 17.0 was used for all statistical analyses (StataCorp., College Station, TX, USA). Network meta-analysis combines direct and indirect evidence to compare multiple interventions within one treatment network. A random-effects network meta-analysis was performed to cognitive outcomes across different exercise modalities. Treatment effects were reported as SMDs with 95% CIs. Network plots showed the connections among treatments, while forest plots presented the effect estimates for each intervention. The relative effectiveness of interventions was evaluated using the surface under the cumulative ranking curve (SUCRA) (Salanti et al., 2011). SUCRA scores were reported as percentages. Higher scores indicated a higher likelihood of a better rank. To account for multiple testing, Holm’s step-down adjustment was applied separately within each outcome to all active intervention-versus-placebo comparisons. Holm-adjusted *p* values below 0.05 were considered statistically significant.

In addition, transitivity was evaluated separately for each outcome by comparing potential effect modifiers across intervention groups. These included mean age, baseline cognitive or clinical status, intervention length, weekly frequency, session length, and comparator type. Continuous variables were reported as medians and interquartile ranges and compared using Kruskal–-Wallis tests. Categorical variables were reported as frequencies and percentages and compared using permutation chi-square tests. These tests provided supporting evidence only. The final assessment also considered distributional overlap, clinical similarity, node size, and missing data. Full results are provided in Supplementary Material File S6.

### 2.6. Assessment of Heterogeneity and Publication Bias

We assessed heterogeneity using the I^2^ and τ^2^ statistics. An I^2^ value above 50% indicated moderate or high heterogeneity. Publication bias was assessed using funnel plots. Egger’s test was conducted when sufficient studies were available. This test was used to detect possible small-study effects.

### 2.7. Quality Assessment

Independent risk-of-bias assessments were completed by two authors using the Cochrane RoB 2 tool. A third reviewer helped resolve any disagreements. The risk of bias assessment covered five domains: (1) bias arising from the randomization process; (2) bias due to deviations from intended interventions; (3) bias due to missing outcome data; (4) bias in measurement of the outcome; (5) bias in selection of the reported result.

Blinding participants and intervention providers is often not possible in exercise trials. Therefore, lack of blinding alone was not automatically considered a high risk of bias. Following RoB 2 guidance, Domain 2 assessed whether knowledge of group assignment caused deviations from the intended intervention. Domain 4 assessed whether this knowledge could influence outcome measurement.

## 3. Results

**Figure 1:**
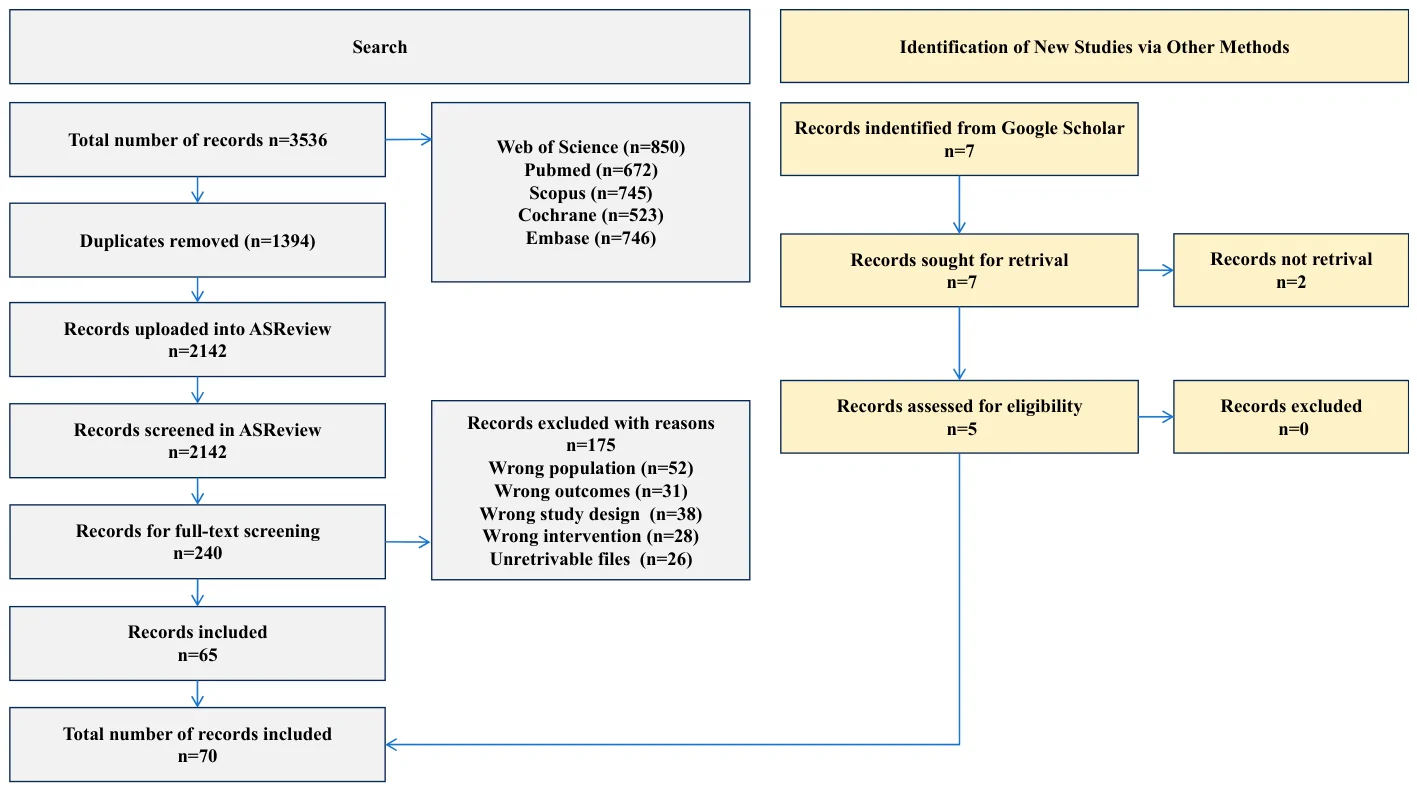
Study flow diagram.

The database search identified 3536 records: 850 from Web of Science, 672 from PubMed, 745 from Scopus, 746 from Embase, and 523 from the Cochrane Library. After 1394 duplicates were removed, 2142 records underwent title and abstract screening, followed by full-text review. Finally, the network meta-analysis included 70 RCTs with a total of 5573 participants. Studies were mainly excluded because of unsuitable study designs (*n* = 52), irrelevant outcomes (*n* = 31), ineligible interventions (*n* = 38), non-target populations (*n* = 28), or inappropriate control groups (*n* = 26). The study selection process is shown in Figure 1. The complete list of included studies is provided in Supplementary Material File S3.

**Figure 2:**
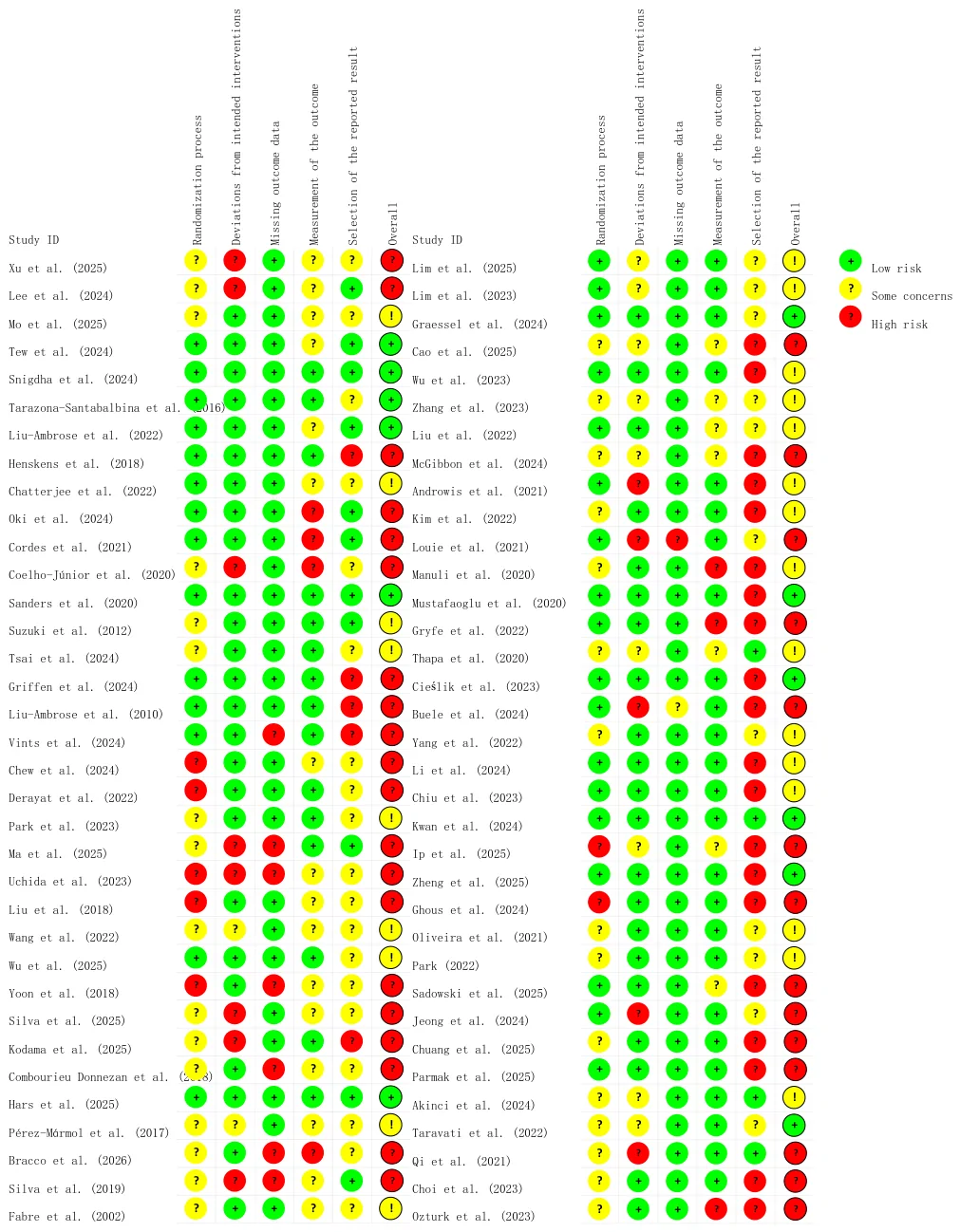
Risk of bias in the included RCTs was assessed using the Cochrane tool. Each row represents one study and shows judgments across six domains. D1: Random sequence generation. D2: Allocation concealment. D3: Blinding of participants and personnel. D4: Blinding of outcome assessment. D5: Incomplete outcome data. Overall: Overall risk of bias judgment. Full details of RCTs are provided in Supplementary File S3.

The included studies demonstrated good methodological quality (Figure 2). Most studies had a low risk of bias in randomization and missing outcome data. Some concerns were identified mainly in deviations from intended interventions and outcome measurement, largely reflecting the difficulty of full blinding in exercise intervention trials. Most studies were rated as having low risk or some concerns, while only a small number had high risk. Overall, the evidence was considered reasonably robust.

### 3.1. Cognitive Function

**Figure 3:**
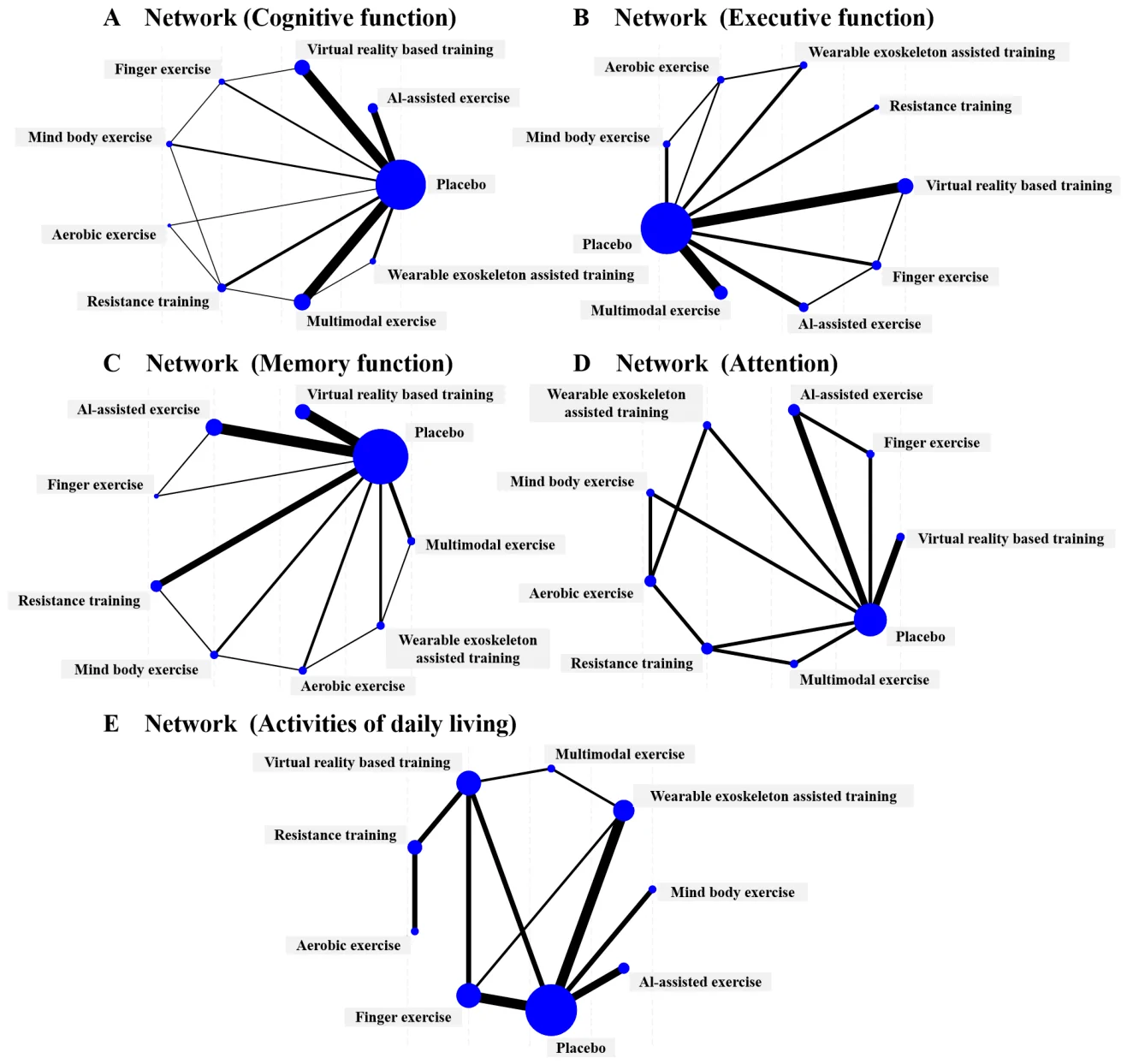
Network diagrams comparing interventions across five health outcomes in older adults: (**A**) cognitive function, (**B**) executive function, (**C**) memory function, (**D**) attention, and (**E**) activities of daily living (ADL). Each node represents a type of intervention: Al-assisted exercise, Wearable exoskeleton-assisted training, Virtual reality-based training, Mind–body exercise, Aerobic exercise, Resistance training, Multimodal exercise, Finger exercise, and Placebo. Larger nodes represent interventions with more participants. Connections between nodes show direct comparisons. Line thickness increases with the number of RCTs available for each comparison.

Global cognitive performance was assessed in 41 randomized controlled trials involving 2107 participants. The primary outcome of global cognition was assessed using network meta-analysis, with results presented in Figure 3A. The most commonly used assessment instruments for global cognition were the Korean version of the Mini-Mental State Examination (K-MMSE) (n = 21, 51.21%) and the Montreal Cognitive Assessment (MoCA) (n = 15, 36.59%).

**Figure 4:**
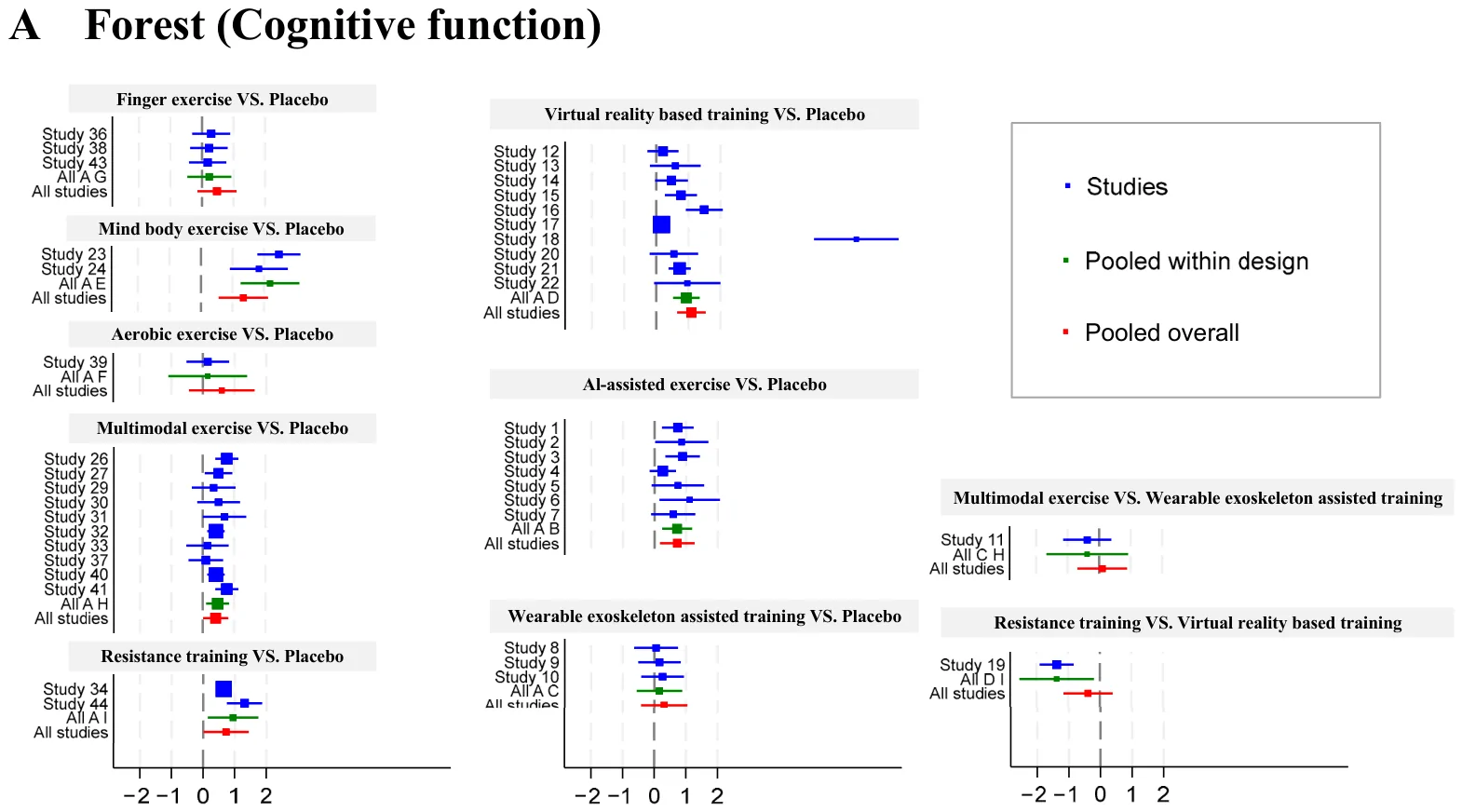

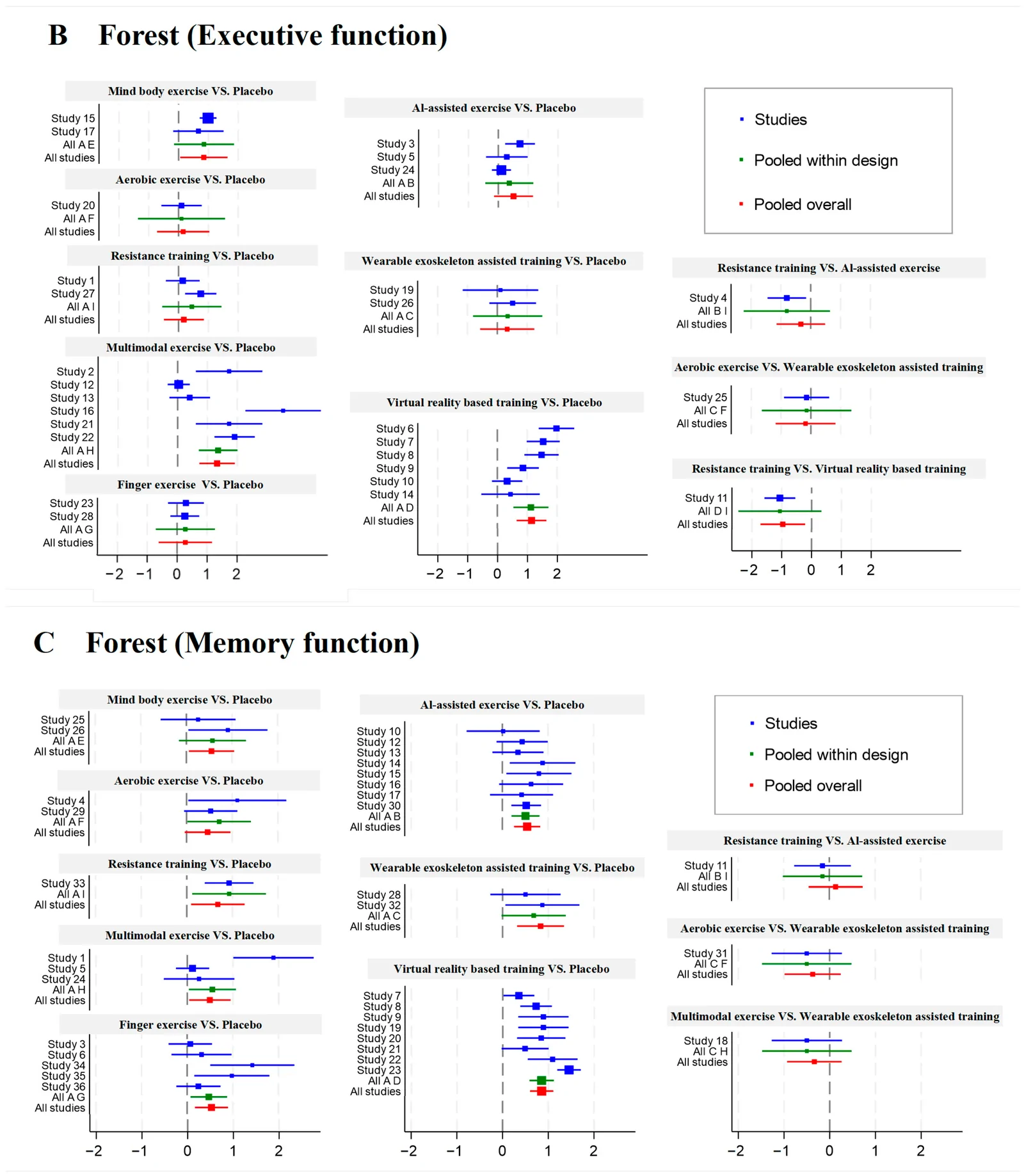

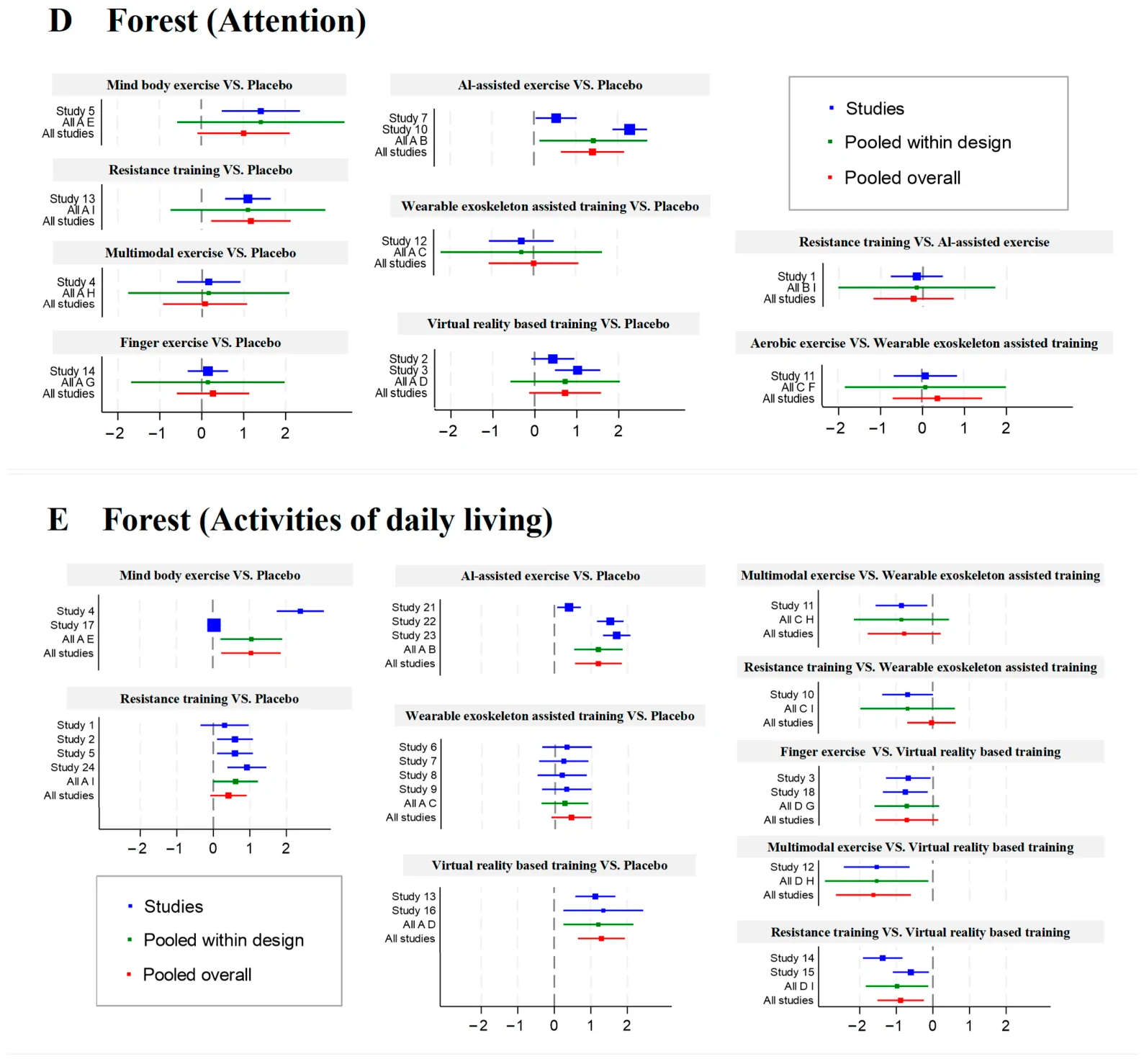
Forest plots showing network meta-analysis results for eight intervention types (Al-assisted exercise, Wearable exoskeleton-assisted training, Virtual reality-based training, Mind–body exercise, Aerobic exercise, Resistance training, Multimodal exercise, Finger exercise, placebo) across five key outcomes in older adults: (**A**) Cognitive function, (**B**) Executive function, (**C**) Memory function, (**D**) Attention, and (**E**) Activities of daily living (ADL). Treatment effects are reported as SMDs and their corresponding 95% CIs. For each outcome, individual study estimates are first displayed for the corresponding direct comparisons. The “pooled within-design” estimate refers to the pooled effect calculated from studies with the same direct comparison or study design. The “pooled overall” estimate represents the final network-derived SMD for each specific comparison, obtained by combining direct and indirect evidence under the random-effects consistency model, rather than a simple average across studies or interventions.

**Figure 5:**
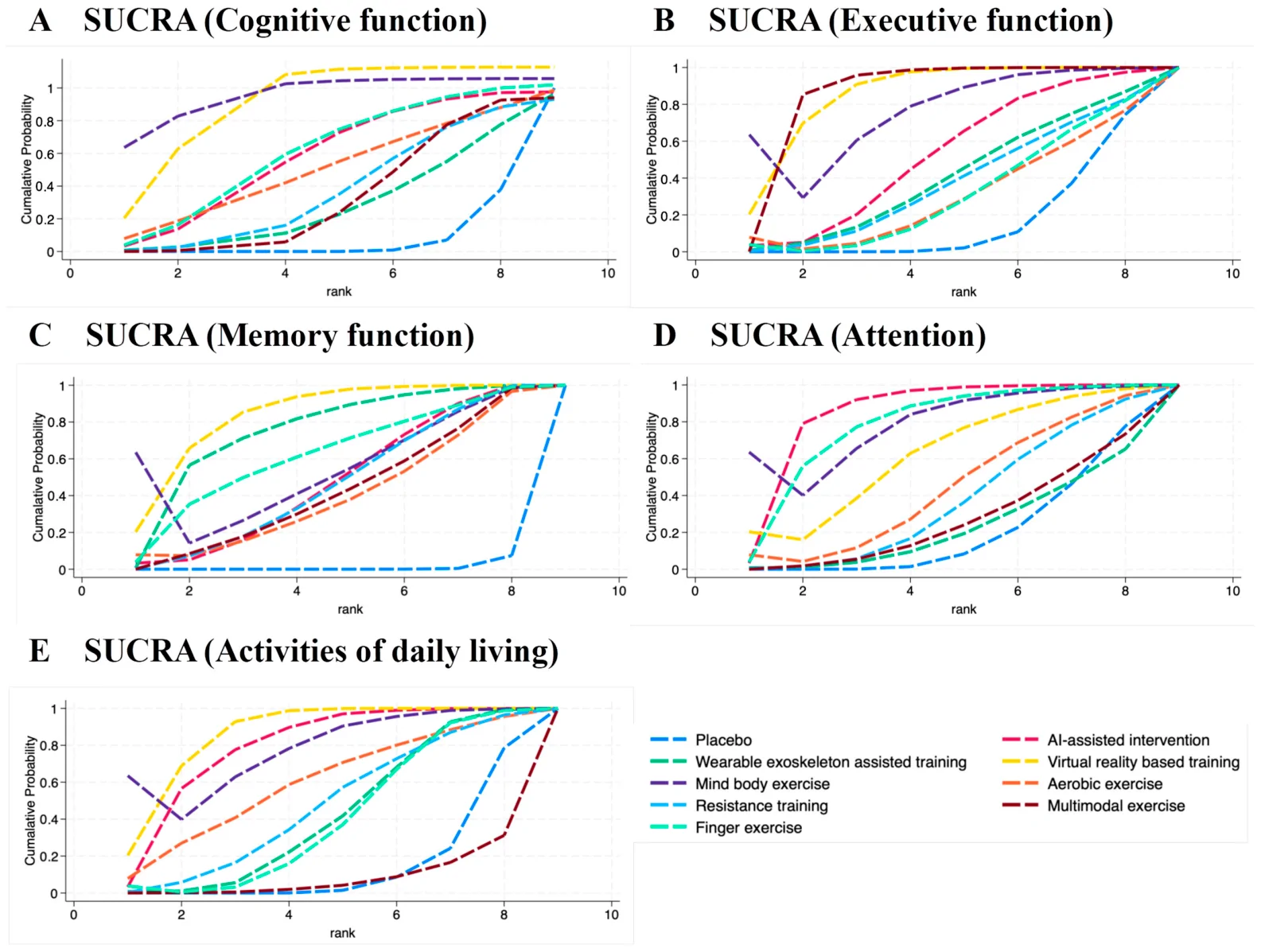
Cumulative ranking probability plots based on SUCRA for five outcomes. Each panel shows how the interventions were ranked for one outcome: (**A**) Cognitive function, (**B**) Executive function, (**C**) Memory function, (**D**) Attention, and (**E**) Activities of daily living (ADL). SUCRA shows the likelihood that each intervention will achieve a higher rank. A more favorable rank is indicated when the curve approaches the upper-left corner.

The network meta-analysis (NMA) showed that, compared with conventional rehabilitation or placebo control, all active interventions demonstrated effects in a favorable direction on global cognitive outcomes (Figure 4A). As shown in Figure 5A, mind–body exercise had the highest SUCRA ranking and showed a significant effect on global cognition (SMD = 2.19, 95% CI: 1.26–3.12, *p* < 0.001). After Holm correction, the effects of mind–body exercise and virtual reality-based exercise remained statistically significant (Supplementary Material File S5). Virtual reality-based exercise also exhibited substantial benefits for global cognition (SMD = 1.09, 95% CI: 0.65–1.54). Both AI-assisted exercise and finger exercise demonstrated positive effects on cognitive performance. Although statistical significance was less certain in some pairwise comparisons, these interventions achieved relatively high ranking probabilities (AI-assisted exercise SUCRA = 58.2%; finger exercise SUCRA = 56.9%).

The network consistency test indicated no significant inconsistency (χ^2^(6) = 12.30, *p* = 0.0556), suggesting overall agreement between direct and indirect evidence. Heterogeneity analyses indicated some variability across studies; however, the overall effect estimates were consistent and robust, supporting the validity of integrated comparison and ranking of intervention effects across different exercise modalities.

### 3.2. Executive Function

Executive function was assessed in 27 randomized controlled trials involving 1531 participants. Executive-related primary outcomes were assessed using network meta-analysis (Figure 3B). The assessment tools used across studies included language-based executive tasks (n = 8, 30.77%) and the Digit Symbol Modalities or Substitution Tests (SDMT, SDST, DSST) (n = 5, 19.23%).

The network meta-analysis suggested differences in the estimated effects of the various exercise interventions on executive function in older adults (see Figure 4B). Multimodal exercise (SUCRA = 92.6%, PrBest = 60.2%) and virtual reality-based exercise (SUCRA = 85.7%, PrBest = 25.6%) occupied the top two positions in the SUCRA ranking, and both showed statistically significant effects compared with placebo (multimodal exercise: SMD = 1.32, 95% CI: 0.73–1.92; virtual reality-based exercise: SMD = 1.15, 95% CI: 0.65–1.65). Mind–body exercise (SUCRA = 70.0%) showed a moderate to large improvement in executive function (SMD = 0.85, 95% CI: 0.05–1.64), but this effect did not remain statistically significant after Holm correction (Supplementary Material File S5). AI-assisted exercise yielded an effect size of SMD = 0.51 (95% CI: −0.14 to 1.16) and were not statistically significant. Wearable exoskeleton training, resistance training, aerobic exercise, and finger exercise ranked relatively lower, with most comparisons against placebo failing to demonstrate significant differences. The corresponding SUCRA rankings are shown in Figure 5B.

The network consistency test showed no evidence of inconsistency (χ^2^(4) = 0.83, *p* = 0.934), and between-study heterogeneity was moderate (τ ≈ 0.58), indicating coherence between direct and indirect evidence, although the precision of the estimates varied across comparisons.

### 3.3. Memory Function

Memory outcomes were assessed in 32 randomized controlled trials involving 1912 participants. Primary memory-related data were synthesized using network meta-analysis (Figure 3C). Memory performance was assessed using a range of instruments, including delayed recall from the Chinese Auditory Verbal Learning Test (CAVLT) (n = 4, 22.22%), short-term memory measured by the Paired Memory Test (PMT) (n = 4, 22.22%), verbal immediate and delayed recall from the CVVLT and AVLT (n = 2, 11.11%), the memory subscale of the SCOPA-COG for Parkinson’s disease (n = 2, 11.11%), the Wechsler Memory Scale (WMS) (n = 2, 11.11%), and the Digit Span Forward and Backward tests (DSF) (n = 2, 11.11%).

According to SUCRA-based rankings, virtual reality-based interventions (SUCRA = 84.9%) and wearable exoskeleton training (SUCRA = 78.6%) had the highest relative rankings and were both statistically superior to placebo. The corresponding effect sizes were SMD = 0.85 (95% CI: 0.60–1.11) for virtual reality and SMD = 0.83 (95% CI: 0.32–1.35) for wearable exoskeleton training. Finger exercise ranked third (SUCRA = 62.1%) and was also consisted with a statistically significant improvement in memory performance (SMD = 0.68, 95% CI: 0.09–1.27). Mind–body exercise and AI-assisted exercise occupied intermediate positions in the ranking hierarchy, with SUCRA values of 49.3% and 46.7%, respectively. Both interventions produced small to moderate yet statistically significant benefits in memory function (mind–body exercise: SMD = 0.55, 95% CI: 0.05–1.05; AI-assisted exercise: SMD = 0.54, 95% CI: 0.26–0.83). In contrast, aerobic exercise, resistance training, and multimodal exercise yielded comparatively modest effects, with several comparisons failing to reach statistical significance. After Holm adjustment, virtual reality-based training, AI-assisted exercise, and resistance training remained statistically significant. Wearable exoskeleton training, finger exercise, mind–body exercise, and multimodal exercise were no longer significant. These results suggest differences among interventions. However, the rankings should be interpreted cautiously because evidence for memory outcomes was limited (Supplementary Material File S5). Further details are provided in Figure 4C and Figure 5C.

No significant inconsistency was detected within the network (χ^2^(5) = 2.73, *p* = 0.741), and between-study heterogeneity was low (τ ≈ 0.29), showing consistency between direct and indirect evidence and supporting the reliability of the network estimates.

### 3.4. Attention

Attention outcomes were assessed in 14 randomized controlled trials involving 847 participants. Network meta-analysis was used to combine the primary outcome data (Figure 3D). Attention was assessed using attention-specific scales in six studies (42.86%) and combined attention and calculation tests in four studies (28.57%).

All active interventions showed better outcomes than conventional rehabilitation or placebo, details are shown in Figure 4D. Among the included interventions, AI-assisted exercise had the highest SUCRA-based ranking (SUCRA = 89.1%, PrBest = 46.6%) and was statistically significant compared with placebo (SMD = 1.40, 95% CI: 0.64–2.16). Finger exercise also showed a statistically significant effect (SMD = 1.18, 95% CI: 0.23–2.13) and ranked second (SUCRA = 79.4%, PrBest = 24.1%), but it did not remain statistically significant after Holm correction. Mind–body exercise ranked third (SUCRA = 74.4%, PrBest = 21.2%). However, the attention network included only 14 RCTs, and several confidence intervals were wide. The rankings should be viewed as preliminary rather than firm evidence of superiority. In contrast, wearable exoskeleton training (SUCRA = 22.5%), resistance training (SUCRA = 36.4%), and multimodal exercise (SUCRA = 26.2%) showed comparatively weaker effects on attention outcomes. For details, see Figure 5D.

Overall heterogeneity across studies was moderate (I^2^ = 63.5%, τ = 0.245), indicating some variability in effect estimates that did not substantially compromise interpretability. The consistency assessment revealed no significant inconsistency within the network (χ^2^(4) = 0.48, *p* = 0.975), suggesting consistency within the network, although some estimates remained imprecise because few studies were available.

A univariable random-effects network meta-regression examined whether intervention duration contributed to between-study variation. Intervention duration, measured in weeks, was included as a study-level covariate under a common interaction assumption. The analysis showed that longer intervention duration was associated with greater improvements in attention (β = 0.086 per week, 95% CI: 0.033–0.139, *p* = 0.001). After adjustment for intervention duration, residual heterogeneity decreased from τ^2^ = 0.312 to τ^2^ = 0.077, corresponding to an estimated 75.2% reduction in between-study variance. However, the association was no longer statistically significant after the separate exclusion of two influential studies, indicating that this finding should be interpreted as exploratory.

### 3.5. Activities of Daily Living (Adl)

Twenty-three randomized controlled trials involving 1719 participants evaluated how eight interventions affected activities of daily living (Figure 3E). The most common measures were the IADL scale in six studies (31.58%), the EQ-5D index in four studies (21.05%), and the Barthel Index in two studies (10.53%).

Results from the network meta-analysis indicated that each active intervention was associated with improvements in daily functioning when contrasted with usual care or placebo, as presented in Figure 4E. Among all interventions, virtual reality-based training had the highest SUCRA ranking (SUCRA = 86.9%, PrBest = 34.2%) and showed a statistically significant advantage over placebo (SMD = 1.30, 95% CI: 0.65–1.94). AI-assisted exercise also ranked highly (SUCRA = 81.4%, PrBest = 30.3%) and produced a comparably strong and significant effect on activities of daily living (SMD = 1.20, 95% CI: 0.56–1.84). Improvements of moderate to large magnitude were observed for mind–body exercise (SUCRA = 72.9%; SMD = 1.05, 95% CI: 0.23–1.86), but this effect did not remain statistically significant after Holm correction (Supplementary Material File S5). By contrast, the effect of aerobic exercise was not statistically significant (SMD = 0.81, 95% CI: −0.60–2.21). Interventions involving wearable exoskeletons, resistance training, and finger exercise yielded relatively modest gains, with no significant differences detected in comparison with placebo. The corresponding SUCRA rankings are shown in Figure 5E.

Assessment of network consistency showed no evidence of disagreement between direct and indirect comparisons (χ^2^(3) = 1.77, *p* = 0.621). Although studies showed a moderate level of heterogeneity (τ ≈ 0.54), the overall coherence of the network supported the robustness of the combined estimates.

The distributions of potential effect modifiers across intervention nodes are presented in Supplementary Material File S6. Some imbalances were observed in participant characteristics and intervention dose, particularly in the global cognition, memory, and ADL networks, indicating that transitivity was broadly plausible but not fully assured and that the indirect comparisons should be interpreted cautiously.

## 4. Discussion

Using a unified network meta-analysis framework, this study systematically compared the relative effects of traditional exercise interventions and non-traditional technology-assisted exercise interventions across multiple cognitive outcomes. Overall, our findings align with earlier findings that conventional exercise and virtual reality-based interventions may improve cognition in older adults (H. Han et al., 2025; Wu et al., 2019; Ren et al., 2024; Tortora et al., 2024). However, we suggest that mind–body exercise may be more strongly associated with global cognition, whereas some technology-assisted interventions ranked favorably in selected cognitive and functional domains. The effects varied across cognitive domains. Therefore, the results do not show that one general intervention category is superior in all outcomes.

These observations align closely with the emerging consensus in the existing literature. Accumulating evidence indicates that the cognitive benefits of both mind–body exercise and conventional physical activity are more robustly manifested in the maintenance of global cognitive functioning and attenuation of age-related functional decline, with effects largely dependent on sustained behavioral engagement, rather than rapid or domain-specific cognitive enhancement (Kaufman et al., 2024). By comparison, interventions based on virtual reality and intelligent feedback systems are often characterized by increased task complexity, explicit goal-oriented structures, and real-time adaptive feedback (Wang et al., 2025). Such features are likely to exert more direct stimulation on executive control, attentional allocation, and memory processing, thereby producing larger effect sizes in these specific domains. A key contribution of the present study lies in demonstrating that this differentiation is not incidental. By integrating evidence from multiple trials within a single analytical framework, the results show that the observed pattern remains directionally consistent after evidence synthesis. This finding suggests that traditional exercise interventions and technology-assisted approaches may influence cognitive function in older adults through partially distinct pathways.

### 4.1. Potential Mechanisms Underlying the Cognitive Benefits of Mind–Body Exercise

Mind–body exercise may influence cognitive function primarily through sustained modulation of autonomic balance and stress-related neurophysiological pathways, which are closely linked to attentional control, emotional regulation, and long-term reorganization of the autonomic nervous system (Daniela et al., 2022). Accumulating evidence indicates that by alleviating stress, improving sleep quality, and reducing anxiety, mind–body practices create favorable conditions for hippocampus-related plastic changes (Mukherjee et al., 2024). In parallel, mind–body practices involving attentional engagement and breath regulation support coordinated prefrontal-limbic regulation, thereby contributing to emotional stability and self-regulatory control (Rathore et al., 2022; Zaccaro et al., 2018). With continued practice, improvements in bodily control are accompanied by enhanced coordination of sensory and motor processes, supporting more stable postural control, motor coordination, and balance regulation (Kaufman et al., 2024; Zaccaro et al., 2018).

From a neurobiological perspective, the effects of long-term practice are not confined to isolated brain regions but are reflected in enhanced interregional functional connectivity and more coordinated patterns of neural activity across distributed brain systems (Rathore et al., 2022; Zaccaro et al., 2018). The motor and sensorimotor cortices engaged during whole-body coordinated movement training contribute to sensorimotor integration and coordinated limb movements (Demirakca et al., 2016), while the prefrontal cortex remains engaged in attention regulation, inhibitory control, and the top-down modulation of emotional processing (Friedman & Robbins, 2022). The hippocampus and related limbic structures contribute to the regulation of stress responses and are closely linked to plastic changes associated with memory processes (Y. M. Y. Han et al., 2023). Neuroimaging studies further demonstrate that mind–body exercise induces systematic alterations in brain structure, functional connectivity, and neural activity patterns (Y. M. Y. Han et al., 2023; Yue et al., 2020). Consequently, these mechanisms may help explain why mind–body exercise showed favorable effects on global cognition. However, whether these benefits reflect stable neuroplastic changes and can be maintained over time requires confirmation through studies with longer follow-up periods.

### 4.2. Potential Mechanisms Underlying the Cognitive Benefits of Technology Assisted Interventions

Non-traditional interventions such as virtual reality, artificial intelligence, and wearable exoskeletons tend to preferentially enhance task-oriented capacities in older adults, particularly in domains related to executive control, decision-making, and perceptual-motor integration. By embedding training within highly complex and explicitly goal-directed task environments, these interventions impose a relatively high cognitive load and provide strong stimulation to the aging cognitive system (Barry et al., 2014). Although overall neural plasticity declines with advancing age, targeted high-demand tasks can still elicit plastic responses in functionally relevant neural pathways, especially those supporting executive and attentional processes (Anguera et al., 2022). At the same time, AI-driven training systems provide real-time feedback and adaptive difficulty adjustment, facilitating timely error correction and dynamic strategy updating during learning, which may accelerate improvements in specific cognitive functions (Strielkowski et al., 2025).

At the neural level, immersive technologies create favorable conditions for coordinated activation across multiple brain regions (Y. M. Y. Han et al., 2023). Spatial navigation and contextual memory tasks in virtual reality strongly recruit the hippocampus, which is central to spatial and episodic memory (Eichenbaum, 2017). By contrast, cognitive training components such as goal maintenance, task switching, inhibitory control, and decision-making predominantly involve prefrontal cortical networks (Friedman & Robbins, 2022). When technological interventions incorporate bodily movement or interactive motor components, such as virtual walking, limb manipulation, or exoskeleton-assisted exercise, motor and sensorimotor cortical regions are concurrently engaged, supporting the integration between cognitive processing and motor control (Barry et al., 2014). Accumulating evidence suggests that virtual reality-based and immersive 3D training environments are associated with measurable functional and structural brain changes across prefrontal, hippocampal, and motor-related regions, including alterations in neural activation patterns and gray matter volume in analogous virtual learning paradigms (Gangemi et al., 2023; Shen et al., 2023; West et al., 2017). These neural adaptations provide a plausible mechanistic explanation for the favorable effects observed at the end of the intervention in specific cognitive domains following technology-assisted interventions.

### 4.3. Traditional and Technology-Assisted Interventions Confer Complementary Cognitive Benefits Rather than Acting as Direct Substitutes

The study found that different interventions showed different patterns of cognitive benefits. Non-traditional technology-assisted interventions performed better in executive function, attention, and memory. Traditional exercise, especially mind–body exercise, showed greater benefits for global cognition. Therefore, one intervention category should not be considered consistently superior to the other. These differences may be related to intervention duration and training characteristics. Supplementary Material File S3 showed that the mean intervention duration was 8.9 weeks for non-traditional interventions and 17.5 weeks for traditional intervention. Many non-traditional interventions were shorter but involved higher task demands and real-time feedback. These features may help improve specific cognitive functions. However, improvements observed immediately after training may also partly reflect practice or test-familiarity effects. Traditional exercise usually requires repeated and sustained practice. However, long-term follow-up data remain limited. Future studies should extend both intervention and follow-up periods.

Accordingly, traditional and non-traditional interventions should not be viewed as substitutes for each other. Nontraditional interventions may be particularly well suited to early or intensive phases of intervention, where high-intensity, task-oriented training can rapidly activate executive and attentional networks (Wang et al., 2025). Traditional mind–body practices, by contrast, more suitable for continued and repeated practice, consistent with the relatively longer intervention periods observed in the included studies (Rathore et al., 2022). This stage-specific strategy still requires further validation.

Moreover, compared with a single intervention, a stage-specific and complementary strategy may better reflect the characteristics of cognitive plasticity in older adults. Technology-assisted interventions, such as virtual reality, can increase immersion, task engagement, and enjoyment. These features may improve motivation, participation, and adherence (Hutchinson, 2024; Touloudi et al., 2025; Maharjan et al., 2024). Gamified training may also promote social interaction and a sense of achievement, thereby mitigating the adverse effects of social isolation and poor motivation (Kalantari et al., 2023; Koivisto & Malik, 2021). Mind–body exercise may improve basic attention, emotional regulation, and fatigue tolerance. These benefits can provide a psychological and physiological foundation for demanding task-based training (Calderone et al., 2024).Within an integrated framework, technology-based interventions may support executive functions through complex task engagement, whereas mind–body practices may support cognitive regulation through repeated modulation of attention, emotion, and stress (Friedman & Robbins, 2022; Y. M. Y. Han et al., 2023). Such a combined strategy acknowledges the relative constraints of cognitive plasticity in later life while activating latent capacities through multiple pathways, offering a more nuanced and potentially more effective approach to cognitive enhancement in older adults.

### 4.4. Strengths and Limitations

This study has several notable strengths. First, it adopts a unified network meta-analysis framework to simultaneously compare traditional exercise interventions and technology-assisted exercise interventions, combining evidence from direct and indirect comparisons. This approach enables robust cross-modality comparisons that cannot be achieved through conventional pairwise meta-analyses and provides a more comprehensive evaluation of the relative effectiveness of different intervention strategies. Second, the study extends beyond global cognitive outcomes by systematically examining multiple cognitive and functional domains, including executive function, memory, attention, and activities of daily living. By examining multiple cognitive domains separately, this approach clarifies which exercise types may benefit specific functions in older adults and helps guide more tailored interventions. Finally, by revealing differentiated patterns of cognitive benefits between traditional and technology-assisted interventions, the findings provide empirical support for stage-specific and integrative intervention strategies. Rather than viewing these approaches as interchangeable alternatives, the results highlight their complementary roles in cognitive aging, offering important theoretical and practical implications for optimizing non-pharmacological interventions across different stages of later life.

Several limitations should be considered. First, substantial variability existed in the implementation of different intervention technologies. Differences in VR device types, levels of immersion, weekly training frequency, session duration, and interaction design may have increased heterogeneity. Similarly, AI-assisted exercise also included systems with different technical mechanisms, reducing the specificity of this category. Second, intervention durations varied considerably, and most outcomes were assessed immediately after treatment. Thus, short-term improvements may partly reflect practice effects rather than stable neuroplastic changes, while limited follow-up data preclude conclusions about their long-term persistence. Third, outcome measures varied across cognitive domains, which may have introduced systematic bias into effect estimation. Fourth, relatively small sample sizes reduced the robustness of statistical inference. SUCRA rankings may accentuate apparent differences between interventions when effect estimates are imprecise or overlapping. Finally, the lack of blinding in some studies increased the risk of bias.

## 5. Conclusions and Future Perspectives

In conclusion, both traditional exercise and technology-assisted exercise interventions are beneficial for cognitive health in older adults, although their effects differ across cognitive domains. Traditional exercise, particularly mind–body exercise, showed favorable effects on global cognition, whereas virtual reality-based and other technology-assisted interventions showed potential advantages in selected cognitive and functional outcomes. These findings indicate that traditional and technology-assisted exercise interventions may support cognitive health through partially distinct yet complementary pathways in aging populations, but their comparative effects require further confirmation in well-designed studies.

Future research should improve study design and strengthen the evidence base. More standardized intervention protocols are needed. These protocols should clearly define intervention content, intensity, and technology use. This would improve comparisons across studies. Current evidence shows that traditional exercise interventions have been examined over relatively longer periods than many technology-assisted interventions and long-term follow-up data remain limited for both categories. This imbalance in temporal evidence makes it difficult to determine the true long term potential of technology-assisted interventions and suggests that their sustained cognitive effects may not yet be fully captured. Future studies should examine technology-assisted interventions over longer treatment and follow-up periods. They should also use rigorous randomized designs, larger samples, and multicenter settings. Stronger and more reproducible evidence is needed to clarify long-term effects, possible mechanisms, and real-world value. Such evidence may better support clinical practice and health policy for older adults.

## Data Availability

The data supporting the findings of this study are available within the article and its Supplementary Materials. No new data were created.

## Supplementary Materials

The following supporting information can be downloaded at: https://www.mdpi.com/article/10.3390/jintelligence14080177/s1. File S1: Search strategies, operational definitions, intervention classification, and ASReview-assisted screening process; File S2: Group-level data for global cognition, executive function, memory, attention, and activities of daily living; File S3: Characteristics of the included studies; File S4: PRISMA 2020 checklist; File S5: Holm-adjusted P values for active intervention versus placebo comparisons; File S6: Transitivity assessment of prespecified potential effect modifiers across intervention nodes. Reference (Page et al., 2021) is cited in the supplementary materials.
