# Supplementary material for: Effects of Traditional and Technology-Based Exercise Interventions on Cognitive Function in Older Adults: A Systematic Review and Meta-Analysis
Source: J Intell. 2026 Aug 1;14(8):177. doi: 10.3390/jintelligence14080177 (PMC13514244; doi:10.3390/jintelligence14080177)
Supplement: Supplementary file 1 [file jintelligence-14-00177-s001.zip › S1 Search strategies_V2.pdf]

Effects of Traditional and Technology-Based Exercise Interventions on Cognitive Function in Older Adults: A Systematic Review and Meta-Analysis

1. Search strategies for different databases

(1) Pubmed

(("Exercise"[Mesh] OR "Motor Activity"[Mesh] OR "Rehabilitation"[Mesh] OR exercise[tiab] OR "physical activity"[tiab] OR "aerobic exercise"[tiab] OR "resistance training"[tiab] OR "strength training"[tiab] OR "mind-body exercise"[tiab] OR "tai chi"[tiab] OR qigong[tiab] OR yoga[tiab] OR "finger exercise"[tiab] OR "multicomponent exercise"[tiab] OR rehabilitation[tiab] OR "upper limb rehabilitation"[tiab] OR "lower limb rehabilitation"[tiab] OR "virtual reality"[tiab] OR "Virtual Reality"[Mesh] OR "virtual reality based exercise"[tiab] OR "virtual reality assisted exercise"[tiab]
OR "software assisted rehabilitation"[tiab] OR "technology assisted exercise"[tiab] OR "intelligent rehabilitation"[tiab] OR "adaptive training"[tiab] OR "real-time feedback"[tiab])AND ("Aged"[Mesh] OR "Aged, 80 and over"[Mesh] OR "older adults"[tiab] OR elderly[tiab] OR older[tiab] OR seniors[tiab] OR "aging population"[tiab])AND("Cognition"[Mesh] OR "Cognitive Dysfunction"[Mesh] OR "Executive Function"[tiab] OR "Memory"[Mesh] OR "Attention"[Mesh] OR "Activities of Daily Living"[Mesh] OR cognition[tiab] OR "cognitive function"[tiab] OR "global cognition"[tiab] OR "executive function"[tiab] OR memory[tiab] OR attention[tiab] OR "activities of daily living"[tiab] OR ADL[tiab])AND ("Randomized Controlled Trial"[Publication Type] OR "Controlled Clinical Trial"[Publication Type] OR randomized[tiab] OR randomised[tiab] OR randomly[tiab] OR RCT[tiab] OR trial[tiab]))

(2) Embase

(('exercise'/exp OR 'physical activity'/exp OR 'rehabilitation'/exp OR exercise:ti,ab,kw OR 'physical activity':ti,ab,kw OR 'aerobic exercise':ti,ab,kw OR 'resistance training':ti,ab,kw OR 'strength training':ti,ab,kw OR 'mind-body exercise':ti,ab,kw OR 'tai chi':ti,ab,kw OR qigong:ti,ab,kw OR yoga:ti,ab,kw OR 'finger exercise':ti,ab,kw OR 'multicomponent exercise':ti,ab,kw OR rehabilitation:ti,ab,kw OR 'upper limb rehabilitation':ti,ab,kw OR 'lower limb rehabilitation':ti,ab,kw OR 'virtual reality'/exp OR 'virtual reality':ti,ab,kw OR 'virtual reality based exercise':ti,ab,kw OR 'virtual reality assisted exercise':ti,ab,kw OR 'software assisted rehabilitation':ti,ab,kw OR 'technology assisted exercise':ti,ab,kw OR 'intelligent rehabilitation':ti,ab,kw OR 'adaptive training':ti,ab,kw OR 'real-time feedback':ti,ab,kw)AND('aged'/exp OR elderly:ti,ab,kw OR 'older adult':ti,ab,kw OR 'older adults':ti,ab,kw OR older:ti,ab,kw OR seniors:ti,ab,kw OR 'aging population':ti,ab,kw)AND('cognition'/exp OR 'cognitive defect'/exp OR 'memory'/exp OR 'attention'/exp OR 'activities of daily living'/exp OR cognition:ti,ab,kw OR 'cognitive function':ti,ab,kw OR 'global cognition':ti,ab,kw OR 'executive function':ti,ab,kw OR memory:ti,ab,kw OR attention:ti,ab,kw OR 'activities of daily living':ti,ab,kw OR ADL:ti,ab,kw)AND
('randomized controlled trial'/exp OR 'controlled clinical trial'/exp OR randomized:ti,ab,kw OR randomised:ti,ab,kw OR randomly:ti,ab,kw OR rct:ti,ab,kw OR trial:ti,ab,kw))AND [embase]/lim

(3) Cochrane Library

((MeSH descriptor: [Exercise] explode all trees OR MeSH descriptor: [Motor Activity] explode all trees OR MeSH descriptor: [Rehabilitation] explode all trees OR MeSH descriptor: [Virtual Reality] explode all trees OR exercise:ti,ab,kw OR "physical activity":ti,ab,kw OR "aerobic exercise":ti,ab,kw OR "resistance training":ti,ab,kw OR "strength training":ti,ab,kw OR "mind-body exercise":ti,ab,kw OR "tai chi":ti,ab,kw OR qigong:ti,ab,kw OR yoga:ti,ab,kwOR "finger exercise":ti,ab,kw OR "multicomponent exercise":ti,ab,kw OR rehabilitation:ti,ab,kw OR "upper limb rehabilitation":ti,ab,kw OR "lower limb rehabilitation":ti,ab,kw OR "virtual reality":ti,ab,kw OR "virtual reality based exercise":ti,ab,kw OR "virtual reality assisted exercise":ti,ab,kw OR "software assisted rehabilitation":ti,ab,kw OR "technology assisted exercise":ti,ab,kw OR "intelligent rehabilitation":ti,ab,kw OR "adaptive training":ti,ab,kw OR "real-time feedback":ti,ab,kw) AND (MeSH descriptor: [Aged] explode all treesOR "older adults":ti,ab,kw OR elderly:ti,ab,kw OR older:ti,ab,kw OR seniors:ti,ab,kw OR "aging population":ti,ab,kw) AND (MeSH descriptor: [Cognition] explode all trees OR MeSH descriptor: [Memory] explode all trees OR MeSH descriptor: [Attention] explode all trees OR MeSH descriptor: [Activities of Daily Living] explode all trees OR cognition:ti,ab,kw OR "cognitive function":ti,ab,kw OR "global cognition":ti,ab,kw OR "executive function":ti,ab,kw OR memory:ti,ab,kw OR attention:ti,ab,kw OR "activities of daily living":ti,ab,kw OR ADL:ti,ab,kw) AND (randomized:ti,ab,kw OR randomised:ti,ab,kw OR randomly:ti,ab,kw OR RCT:ti,ab,kw OR trial:ti,ab,kw))

(4) Web of Science

TS = ((exercise OR "physical activity" OR "aerobic exercise" OR "resistance training" OR "strength training" OR "mind-body exercise" OR "tai chi" OR qigong OR yoga OR "finger exercise" OR "multicomponent exercise" OR rehabilitation OR "upper limb rehabilitation" OR "lower limb rehabilitation" OR "virtual reality" OR "virtual reality based exercise" OR "virtual reality assisted exercise" OR "software assisted rehabilitation" OR "technology assisted exercise" OR "intelligent rehabilitation" OR "adaptive training" OR "real-time feedback")AND("older adults" OR elderly OR older OR seniors OR "aging population")AND(cognition OR "cognitive function" OR "global cognition" OR "executive function" OR memory OR attention OR "activities of daily living" OR ADL)AND("randomized controlled trial" OR "controlled clinical trial" OR randomized OR randomised OR randomly OR RCT OR trial))

(5) Scopus

(TITLE-ABS-KEY(exercise OR "physical activity" OR "aerobic exercise" OR "resistance training" OR "strength training" OR "mind-body exercise" OR "tai chi" OR qigong OR yoga OR "finger exercise" OR "multicomponent exercise" OR rehabilitation OR "upper limb rehabilitation" OR "lower limb rehabilitation" OR "virtual reality" OR "virtual reality based exercise" OR "virtual reality assisted exercise" OR "software assisted rehabilitation" OR "technology assisted exercise" OR "intelligent rehabilitation" OR "adaptive training" OR "real-time feedback") AND TITLE-ABS-KEY("older adults" OR elderly OR older OR seniors OR "aging population")AND TITLE-ABS-KEY(cognition OR "cognitive function" OR "global cognition" OR "executive function" OR memory OR attention OR "activities of daily living" OR ADL) AND

TITLE-ABS-KEY("randomized controlled trial" OR "controlled clinical trial" OR randomized OR randomised OR randomly OR RCT OR trial))

2. Search strategy statement:

In this review, AI-assisted exercise referred to exercise or rehabilitation interventions that used AI-related technologies, algorithm-supported systems, or intelligent feedback mechanisms to automatically adjust training tasks, intensity, difficulty, progression, or feedback according to participant performance. AI-assisted exercise was used as a broad functional category. Some included interventions used rule-based, threshold-based, or predefined adaptive algorithms, which are not equivalent to machine learning or deep learning in the strict technical sense. These systems were included because they provided automated feedback or individualized training adjustment based on participant performance. Interventions were included in this category if they met at least one of the following criteria: (1) automatic adjustment of training tasks, intensity, difficulty, or progression based on participant performance data; (2) provision of immediate feedback, error correction, or strategy guidance; (3) partial automated decision support during training, rather than a fully fixed manually predefined protocol.

Virtual reality assisted exercise interventions were defined as exercise or rehabilitation programs that employ virtual reality technology to construct interactive simulated environments, in which participants engage in virtual tasks through actual physical movements. These interventions were required to meet all of the following conditions: (1) exercise tasks or scenarios were presented within a virtual environment; (2) participants interacted with the virtual environment in real time through bodily movements, postural control, or gait related actions; and (3) the virtual environment played a central role in the training process, rather than serving solely as a background display or auxiliary stimulus.

3. Definition and classification of exercise-based interventions

To further clarify the distinction between exercise-based interventions and cognitive therapy, we applied the following operational criteria. An intervention was classified as exercise-based only when its core component involved structured physical movement, motor training, or body-based rehabilitation. These included traditional exercise modalities such as aerobic exercise, resistance training, mind-body exercise, finger exercise, and multicomponent exercise, as well as technology-assisted modalities such as virtual reality-based motor training, artificial intelligence-assisted exercise training, software-guided rehabilitation, and wearable exoskeleton-assisted training.

Interventions were excluded if their primary component was cognitive therapy or cognitive training alone, such as memory strategy training, computerized cognitive tasks, cognitive stimulation, psychological counseling, or cognitive rehabilitation without a clearly defined physical exercise or motor-training component. For technology-assisted interventions, studies were included only when the technology was used to support, guide, monitor, or adapt active physical movement, balance or gait training, limb training, or interactive exercise tasks. Therefore,

the presence of digital technology itself was not considered sufficient for classification as an exercise intervention.

For interventions combining physical and cognitive elements, classification was based on the dominant intervention component, the stated intervention goal, and the actual training procedures described in the original study. Studies were retained when cognitive tasks were embedded within an exercise or motor-training protocol, such as dual-task exercise, VR-based movement training, or AI-guided adaptive exercise. By contrast, studies were excluded when physical activity was absent, incidental, or merely used as a background activity while the main therapeutic component was cognitive training.

4. ASReview-assisted screening process

After duplicate records were removed, the remaining records were imported into ASReview for title and abstract screening. ASReview was used as a screening assistance tool to prioritize potentially relevant records during the initial screening stage. It was not used to automatically determine study eligibility or replace reviewer judgment. Two reviewers screened the records according to the predefined inclusion and exclusion criteria, and potentially eligible studies were then assessed by full-text review. Final inclusion and exclusion decisions were made manually. Any disagreements were resolved through discussion, and a third reviewer was consulted when consensus could not be reached.

5. Classification of Intervention Categories

**Intervention category**	**Included intervention types**
AI-assisted exercise	AI-based rehabilitation systems; intelligent exercise platforms; adaptive exercise systems; automated feedback-based training
Wearable exoskeleton-assisted training	Lower-limb exoskeleton training; robotic-assisted gait training; wearable robotic rehabilitation devices
Virtual reality-based training	Immersive VR exercise; non-immersive VR training; VR balance training; VR-based cognitive-motor training; VR rehabilitation games
Mind-body exercise	Tai Chi; Baduanjin; Qigong; Yoga; mindfulness-based movement practices
Aerobic exercise	Walking training; treadmill exercise; cycling; aerobic dance; moderate-intensity aerobic programs
Resistance training	Elastic band exercise; strength training; weight training; progressive resistance exercise
Multimodal exercise	Combined aerobic and resistance training; aerobic + balance training; combined cognitive-physical exercise programs
Finger exercise	Finger movement training; hand dexterity exercise; finger-based cognitive-motor exercises
